# The clinical utility of molecular diagnostic testing for primary immune deficiency disorders: a case based review

**DOI:** 10.1186/1710-1492-6-12

**Published:** 2010-06-08

**Authors:** Rohan Ameratunga, See-Tarn Woon, Katherine Neas, Donald R Love

**Affiliations:** 1 Department of Clinical Immunology Auckland City Hospital, Park Rd, Grafton, Auckland New Zealand; 2 LabPlus, Auckland City Hospital, Park Rd, Grafton, Auckland New Zealand; 3 Central and Southern Regional Genetic Services, Wellington Hospital, Private Bag 7902, Wellington, New Zealand

## Abstract

Primary immune deficiency disorders (PIDs) are a group of diseases associated with a genetic predisposition to recurrent infections, malignancy, autoimmunity and allergy. The molecular basis of many of these disorders has been identified in the last two decades. Most are inherited as single gene defects. Identifying the underlying genetic defect plays a critical role in patient management including diagnosis, family studies, prognostic information, prenatal diagnosis and is useful in defining new diseases. In this review we outline the clinical utility of molecular testing for these disorders using clinical cases referred to Auckland Hospital. It is written from the perspective of a laboratory offering a wide range of tests for a small developed country.

## Introduction

Primary immune deficiency disorders (PIDs) were first identified in 1952, with the description of agammaglobulinemia by Bruton [1]. In the last few years, the genetic basis of many PID disorders has been identified [2,3]. Most are inherited as single gene defects. Several are X-linked, which accounts for the preponderance of PIDs amongst males. While rare, most are amenable to specific treatment. For example, successful haematopoeitic stem cell transplantation may be curative in patients with severe combined immune deficiency syndrome (SCID). In other PIDs, delayed diagnosis may be associated with disabling complications such as bronchiectasis [4].

Clinical history and physical examination can be helpful in identifying PIDs and the need for further investigation. Once these patients are referred to an immunologist, other tests including vaccine responses can be undertaken [5,6]. Further advanced tests including the enumeration of subsets of lymphocytes using flow cytometry can be useful in evaluating patients [7,8]. For instance, NKT cells may be absent in patients with some forms of X-linked lymphoproliferative disease (XLP), which is caused by mutations in the *SH2D1A *or *XIAP *genes [9]. Elevated double-negative CD4^-^CD8^- ^TCR alpha/beta+ T (DNT) cells are useful markers for autoimmune lymphoproliferative syndrome due to mutations in the *fas *gene [10]. Ultimately, however, identification of the molecular basis of the disorder will secure the diagnosis. We believe molecular diagnostic testing is a critical part of modern patient management and should be regarded as the standard of care.

We have described the development of a customised molecular testing service for PIDs at Auckland City Hospital [11]. We offer full length (Sanger) sequencing with results within a week if the test is established, or two to three weeks for a customised test [11]. In patients with a typical phenotype but normal genomic sequence we offer cDNA sequencing to exclude the possibility of a complex mutation such an inversion or a promoter mutation. To date over twenty different PID genes have been sequenced.

Clinicians have the opportunity to review actual laboratory data and discuss findings with the scientist performing the test. The technical limitations of the scientific findings can be made clear. Proficiency testing is a critical part of genetic analysis. Laboratory errors can have catastrophic consequences for the proband and the family [12]. The service complies with the recent recommendations for quality assurance in laboratories performing molecular diagnostic testing [13] and is accredited by IANZ, the New Zealand laboratory accrediting agency [11]. The model we have described in New Zealand is cost-effective for a developed country with a small population of 4.3 million.

In this review, the value of genetic testing is explored using patient cases referred to the molecular immunology diagnostic service at Auckland Hospital, together with selected examples from the literature.

## The clinical utility of genetic testing in PID disorders

The benefits of PID genetic testing are listed in Appendix 1. This distinction is artificial as the value to patients and families cross these arbitrary boundaries. There are usually multiple indications for genetic testing as illustrated by these cases. Most of the advantages (and disadvantages) described here also apply to other genetic disorders.

### Distinguishing genetic from acquired disorders

Distinguishing congenital from acquired disorders is fundamentally important for patient management. Sometimes drug or virus induced disorders can mimic PIDs. Removal of the causative drug or treating the viral infection may lead to clinical improvement.

Molecular diagnostic tests proved invaluable in characterizing the cellular and molecular pathology of rubella associated Hyper Immunoglobulin M syndrome (rHIM) [14]. Prior to widespread rubella vaccination, cases of dysgammaglobulinemia were described in patients who had suffered congenital rubella. These cases have become very rare since the advent of widespread rubella vaccination. We have had the opportunity to characterize in detail a patient with this disorder.

The patient concerned is 54 years old with elevated polyclonal IgM levels and absent IgA with low levels of IgG. In 1984 before regular immunoglobulin replacement was commenced, he had undetectable IgG. He suffered recurrent lower and upper respiratory tract infections but does not have bronchiectasis in spite of a chronic cough. He has had sinus surgery for chronic rhinosinusitis.

On further questioning he had sensorineural deafness and impaired vision. He wears a hearing aid. His mother was thought to have suffered rubella during pregnancy. Retinoscopy showed typical changes of congenital rubella. The patient was noted to have a persistently elevated rubella IgM titre. His rheumatoid factor was negative indicating the rubella IgM was from *de novo *synthesis. He had normal *in vitro *T cell responses to lectins and antigens. We have previously shown that patients with X linked Hyper Immunoglobulin M syndrome (XHIM) have impaired T cell antigen responses [15]. Examination of his immunophenotype confirmed the presence of B cells bearing surface IgG, consistent with *in vivo *class switching. In contrast to XHIM patients, he was able to generate CD27+ memory B cells [14].

He had normal CD40 ligand expression by flow cytometry. Given his age and relatively good health together with laboratory results, it was felt it was unlikely he had XHIM. This was confirmed by the presence of wild type CD40 ligand sequence. The presence of normal CD40 ligand sequence confirmed that other family members are not at risk of XHIM. It also provided reassurance to the patient who may be at less at risk of complications including lymphoma and liver failure.

Similarly, many drugs are also known to cause hypogammaglobulinemia. We have described a patient with epilepsy who developed profound hypogammaglobulinemia, which completely resolved on stopping his Lamotrigene [16]. In a similar situation, if a mutation was identified in the *BTK *or *SH2D1A *genes, it would indicate the presence of a PID rather than an acquired disorder and would obviate the need to stop critical therapy such as anti-epilepsy drugs.

### Confirming the clinical diagnosis

Genetic testing plays a pivotal in confirming the clinical diagnosis. This is illustrated by the case of an 18 year old male who presented with fulminant infectious mononucleosis in 2006. He suffered hepatic failure and died three days after being transferred to Auckland City Hospital. The history was remarkable in that he had been treated for Burkitt type lymphoma and had made a complete recovery after chemotherapy [17]. Prior to his death, very high levels of EBV DNA (>8 × 10^6 ^copies/ml EBV DNA) were detected in his serum and X-linked lymphoproliferative disorder (XLP) was strongly suspected. Sequencing the *SH2D1A *gene in this patient revealed a point mutation, c.261delT. This mutation causes a translational frameshift and is predicted to result in expression of a truncated protein, thus confirming the diagnosis of XLP.

XLP is a rare disorder characterised by susceptibility to Epstein Barr virus infection [18]. Affected boys have a catastrophic reaction and many die from fulminant infectious mononucleosis. Mutations in the *SH2D1A *[19] and *XIAP *[20] genes have been identified as the cause of these syndromes. The initial study of the proband described above was undertaken in Perth during the time the assay was being developed in Auckland. We have undertaken similar QA studies where mutations in blinded samples have been identified.

### Where presymptomatic diagnosis (at any age) is not possible with protein-based tests

There are no reliable methods to identify presymptomatic XLP patients in the absence of molecular analysis. Immunophenotype and immunoglobulin profiles do not help identify these patients. Flow cytometry can be used to detect the presence and quantity of affected protein in lymphocyte or lymphocyte subsets. Some investigators [21] have found that XLP patients have decreased *SH2D1A *protein expression compared to healthy individuals; however, some patients with XLP have normal SAP (SH2D1A) protein levels [21]. Missense mutations may not abolish protein expression, thereby resulting in false negative results. Mutations of the cytoplasmic tails of cell surface receptors may impair signaling, while allowing cell surface expression of defective proteins. Thus, even when flow cytometry indicates a normal level of cell surface protein, the results must be confirmed by molecular analysis.

### Cascade screening of at-risk relatives

**Figure 1:**
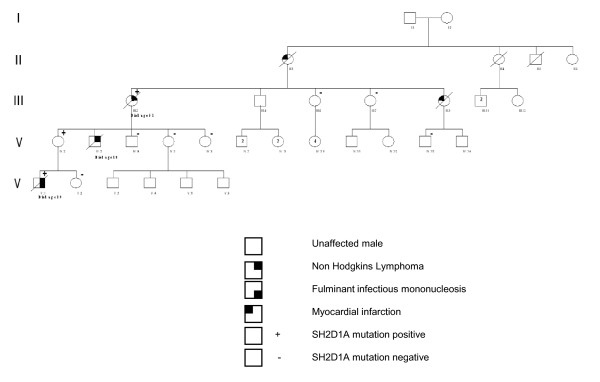
**Pedigree of a family segregating XLP**[17].

Many PID disorders including XLP are inherited in an X-linked fashion. Male patients will manifest the disorder, while females are asymptomatic carriers of the mutation. Figure 1 shows the family tree of this XLP kindred. The male proband (V:1, described above) carries the disease-causing mutation, which was inherited from his mother (IV:1) [17]. Critically, analysis of the proband's sister (V:2) and two maternal aunts (IV:4, IV:5) showed they had not inherited the mutation. Therefore, their children are not at risk of inheriting the disorder. In other disorders, affected members may have phenotypic variation within the same kindred. Genetic testing plays a crucial role in family studies [22].

### Identifying novel presentation of PIDs

A detailed study of this family also revealed that three members had lymphomas before the fatal presentation of the proband [17]. While lymphoma can complicate the course of individual XLP patients, we have suggested that familial lymphoma should be regarded as another presentation of XLP. Supporting evidence comes from Brandau et al [23] who reported *SH2D1A *gene mutations in boys with non-Hodgkins lymphoma, but with no previous EBV infection. Approximately 50% of Hodgkin's and 20% of Burkitt's lymphomas contain EBV DNA [24]. Therefore, it may be prudent to exclude XLP when multiple family members, particularly males, develop lymphoma.

### Identifying atypical presentation of PIDs

During the course of investigation, we found the proband's grandmother (III:1) had died of non-Hodgkins lymphoma at 51 years of age. Lymphoma is one of the classical presentations of XLP in males [25]. We were able to retrieve the grandmother's lymphoma tissue block and extract DNA for further testing. Cloning of the amplified DNA showed that several recombinants contained the mutation, confirming she was a carrier of the disorder [17]. This is the first example of a female who developed an XLP phenotype. More distant members of the kindred may be at-risk and have been advised to seek testing. Again genetic testing played a critical role in confirming symptomatic XLP in a carrier female.

### Characterising the role of molecules in cellular function

Skewed X chromosome inactivation (lyonisation) in symptomatic female carriers of PID genes is well documented [26-29]. In most cases this is a stochastic event where the majority of X chromosomes bearing the wild type allele are inactivated purely by chance. Females may manifest X-linked disorders in this situation. More rarely, skewed lyonisation may be a consequence of mutations at the Xist locus, which initiates X-chromosome inactivation. In this situation the wild type allele may be selectively inactivated in females of the kindred [30].

The identification of a female with an XLP phenotype raised concern that other female carriers including the mother (IV:1) were at risk of symptomatic XLP in this kindred. Analysis of the methylation patterns of the human androgen receptor locus (HUMARA) of the mother (IV:1) did not suggest this family had a disorder of X chromosome inactivation [17]. As a consequence, the most likely explanation for the lymphoma in the affected grandmother (III:1) was skewed X-inactivation. Progressive skewing of lyonisation with aging may place female carriers of X-linked disorders at risk of symptomatic disease [31].

The detection of abnormal lyonisation patterns requires normal tissue. As the paraffin embedded tissue block contained only lymphoma, we were unable to confirm this hypothesis. Our observation suggests that female carriers of a mutation in one copy of the *SH2D1A *gene in other kindreds should be considered at-risk of symptomatic XLP, and hence may need to be monitored for the development of phenotypic features of XLP.

### Assisting treatment decisions

If male children with XLP can be identified before they suffer Epstein-Barr virus infection, hematopoeitic stem cell transplantation can be undertaken which is potentially curative [32-34]. The prognosis after Epstein-Barr virus infection is guarded. In this family, there are no other male patients at risk of XLP.

### Prognosis

Another patient with no family history of recurrent infections presented with a monoarthritis of his knee at the age of 7. At the time he was noted to have absence of tonsils. Testing showed the presence of panhypogammaglobulinemia and immunophenotyping revealed the absence of B cells.

A clinical diagnosis of Bruton's agammaglobulinemia (XLA) was made and the patient was treated with intravenous immunoglobulin (IVIG), even though he did not suffer from frequent or severe infections. The monoarthritis resolved with IVIG treatment, as has been previously described [23]. He has subsequently been in excellent health.

**Figure 2:**
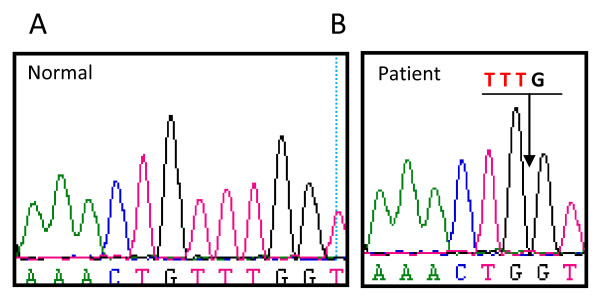
**Electropherogram of the *BTK *gene in a normal donor (A) and patient (B)**. A TGTT deletion in exon 16 leads to a frameshift resulting in a premature stop codon in the *BTK *gene (g.66795_66798delTTGTT, c.1581_1584delTTTG).

Analysis of the patient's DNA revealed the deletion of 4 nucleotides (TTTG) in exon 16 of the *BTK *gene (c.1581_1584delTTTG), which is predicted to cause a frameshift and premature truncation of the btk protein (Figure 2). The molecular basis of the disorder was thus confirmed. As there was no history of recurrent infections, the family was initially uncertain if the patient needed long term IVIG. In this case, mutation analysis confirmed the diagnosis of XLA and the need for life long treatment.

### Early identification of disorders which present later in childhood

The phenotypic manifestations of some disorders are not seen until patients are older. Conventional testing by protein analysis may not be helpful in some situations. This is well-illustrated by type 1 hereditary angioedema (HAE type 1), a disorder caused by autosomal dominant mutations in the *C1NH *gene. Children with this disorder often do not manifest symptoms until adolescence. These patients suffer recurrent angioedema and may be at risk of asphyxia from laryngeal swelling. Complement studies in presymptomatic infants may not be diagnostic even in those who have inherited the mutation [35].

Undertaking genetic studies may enable a presymptomatic diagnosis to be made in the majority of cases, providing prognostic information for the family, and earlier treatment for an affected individual. However, molecular analysis of the *C1NH *gene can be problematic as a significant number of patients have complex mutations including inversions and rearrangements, which can be confirmed by Southern blotting or multiplex fluorescence PCR [36,37]. Genetic diagnosis may not be feasible in all cases of Hereditary angioedema.

### Urgent diagnosis in infancy where conventional diagnostic tests are unreliable

Some PIDs such as XHIM due to CD40 ligand deficiency can prove difficult to confirm in neonates. In normal neonates, CD40 ligand expression in early infancy is reduced and can be difficult to detect by flow cytometry [38]. Therefore, in the case of CD40 ligand deficiency, molecular testing is a more reliable diagnostic option.

**Figure 3:**
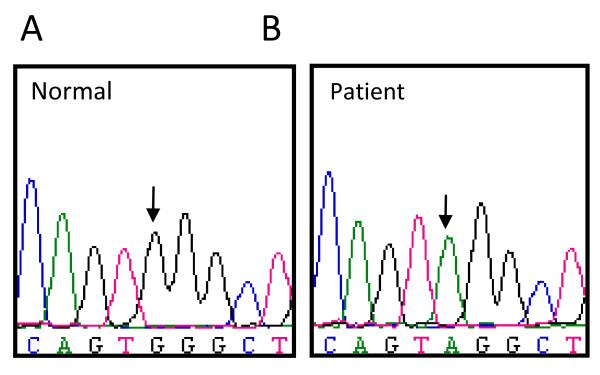
**Electropherogram of the *CD40L *gene in a normal donor (A) and patient (B)**. A point mutation in exon 5 leads to a premature stop codon in the CD40L (c.475G > A, p.W140X).

A 5 month infant was referred to the service with progressive respiratory distress. Bronchoscopy confirmed *Pneumocystis jirovecii *infection. He had an elevated IgM of 1.4 g/l (0.2-1.0) with absent IgG < 0.33 g/l (2.0-7.0) and absent IgA < 0.07 g/l (0.1-0.8) levels. XHIM was suspected. He was treated with Co-trimoxazole and made a complete recovery. Full length sequencing of the CD40 ligand confirmed the presence of a missense mutation (475G > A) leading to a stop codon. (figure 3) In the absence of a suitable bone marrow donor, he has been treated with IVIG and prophylactic antibiotics. He is in good health.

Molecular studies confirmed the mother was a carrier. Subsequently, she gave birth to another son. The region of the (475G > A) mutation was amplified and sequenced. The laboratory was able to confirm that her second child did not carry the mutation.

Given that the familial mutation was known, amplification and sequencing of the specific exon was undertaken within 48 hours. The family was given a definitive diagnosis, which would not have been possible with flow cytometry.

### Prenatal Diagnosis

The identification of a disease-associated mutation offers the possibility of prenatal diagnosis. Prenatal genetic testing requires careful counseling of the family. The counseling should include discussion about the possible outcomes of testing (including the risk of an incorrect result), the risks associated with the procedure, and the options available to the family if an affected fetus is identified. A sample of the fetus' genetic material for such testing is most commonly obtained by chorionic villus sampling (CVS). It is critical that the familial mutation is identified before considering prenatal diagnosis, and that the mother is known to be a carrier of the mutation.

In the case of X-linked disorders, fetal gender is usually determined first as in most cases mutation analysis would only be performed to detect an affected male. PCR studies are undertaken on the sample after maternal tissue contamination is excluded. Prenatal diagnosis enables a couple to identify an affected fetus and then make decisions about the outcome of that pregnancy. If the couple chooses to continue the pregnancy, early treatment of an affected child can occur. In the United Kingdom, prenatal diagnosis is only undertaken if the pregnancy is to be terminated in the case of an affected fetus. Prenatal diagnosis with a rapid turnaround time should be available through a molecular immunology diagnostic service if requested by the family and physicians.

### Pre-implantation Genetic diagnosis

Pre-implantation genetic diagnosis (PGD) is a technique which enables genetic diagnosis of an *in vitro *fertilized embryo before it is implanted into the uterus. This procedure has been undertaken for PIDs [39]. Once an embryo has been created and cultured for between 3 and 5 days, one or more cells are removed at the blastomere or blastocyst stage. DNA is extracted, amplified using PCR, and screened for the familial mutation. If the embryo has not inherited the mutation, it can then be implanted in the uterus.

PGD involves many technical and ethical complexities. Currently the probability of a live-born infant from PGD is approximately 20-25%. This is significantly lower than the conception rate and ongoing pregnancy rate for couples having prenatal diagnosis. However, this technology has many benefits for the couple, and is likely to become more successful in the future. Currently we have a request for this procedure from a family. The many technical and ethical issues need to be carefully considered before this service can be offered.

### Gene therapy

Gene therapy offers the potential to replace a defective gene with a wild type gene. Gene therapy is most likely to succeed in autosomal recessive disorders or X-linked disorders in males. Gene therapy trials have been undertaken for SCID (Adenosine Deaminase deficiency, Common Gamma chain deficiency) and Chronic Granulomatous Disease (CGD) in several countries including the USA, UK, Italy, France and Australia [40,41]. In order to replace the defective gene, the mutation must be identified. Molecular diagnosis thus plays a critical role in any gene therapy trial.

### Assisting with the classification of primary immunodeficiency disorders

The application of molecular techniques has broadened the understanding of PIDs. Seemingly disparate disorders such as Wiskott-Aldrich syndrome and X-linked neutropenia are caused by mutations of the same gene, encoding the Wiskott-Aldrich syndrome protein (WASP) [42]. Allelic heterogeneity, as a result of mutations in different parts of the same gene, can result in varying phenotypic severity or distinct phenotypes as illustrated above. Similarly, phenotypically identical disorders can have an entirely different genetic basis (genocopy). This phenomenon is also known as locus heterogeneity and typically occurs when mutations affect distinct molecules in the same signaling pathway. This is illustrated by agammaglobulinemia, where most male patients have a mutation of the *BTK *gene. However, a similar disorder can be seen in individuals with mutations in the *BLNK *[43] and v λ 5 pre-light chain (*IGLL1*) genes [44]. Similarly, chronic granulomatous disease can be caused by mutations in any of the five genes encoding components of the NADPH oxidase complex (gp91, p47, p21 p40 and p67) [45,46]. Mutation analysis is thus critical in modern disease classification.

### Identification of new genetic defects

Common variable immune deficiency (CVID) is the most frequent symptomatic primary immune deficiency disorder in adults. Patients present with hypogammaglobulinemia, which is associated with an increase in autoimmunity, malignancy and allergy. Approximately 15-20% of patients have a family history of an immune defect in an immediate family member. Over the last five years, four genetic defects have been identified which account for 10-15% of all CVID patients [47-50].

Recently, however, the role of TACI and the BAFF receptor heterozygotes as causes of CVID has been questioned [51]. Many heterozygotes are asymptomatic with no evidence of an immune defect. Many groups are undertaking genetic studies to identify other genes which may be mutated in these disorders. New mutations in some of these CVID patients may lead to reclassification of this group of disorders. Current thinking, however, suggests that CVID may be a polygenic disorder in the majority of affected individuals. High resolution DNA melting analysis [52], whole exome analysis with techniques such as massively parallel sequencing [53] and other novel techniques will accelerate the pace of gene discovery in the future.

### Population based screening for PIDs

Community-based screening tests are well established for disorders such as phenylketonuria, congenital hypothyroidism etc. Recently there has been interest in community screening for Severe Combined Immunodeficiency [54]. This is a rare condition for which effective treatment is available, particularly if identified early. Testing requires extraction of DNA from blood spots from newborn screening cards (Guthrie cards) and the detection of T Cell Receptor Excision Circles (TRECs). While specific defects are not identified by screening, this technology uses similar molecular techniques. The results of these studies are awaited with great interest [55].

## Discussion- the ethics of testing

All case studies described here illustrate the unparalleled power of molecular techniques in solving clinical problems. As indicated above, apart from the examples drawn from our own experience, there are many others in the published literature.

We strongly encourage clinicians to refer their patients for genetic counseling before testing for these disorders. It is very important to discuss the potential advantages and disadvantages of testing with patients before sending blood samples for genetic testing.

The potential for genetic discrimination is widely discussed in the literature. A recent Australian study [56] showed that 10% of over 1000 patients with a variety of genetic diagnoses reported genetic discrimination. The majority of the reported incidents related to life insurance. The article concluded that genetic counseling is essential as genetic professionals have a key role in providing information about the possible negative outcomes of genetic testing in family, social, health and insurance domains.

The technical difficulties in undertaking mutational analysis have been previously described in detail [11,12]. In spite of the power of molecular testing, sometimes the clinical significance of a sequence variation can be difficult to interpret. Molecular testing may add to the complexity of confirming a diagnosis if the nature of the sequence variation is unclear. Other complementary techniques including functional studies may be needed to determine the cellular consequences of a genetic variant of unknown significance. Our own work has shown that even with a classical phenotype, mutations can sometimes be difficult to identify [57]. The causative mutation was identified in only 7 out of 27 patients with suspected PID. Many of 20 undiagnosed patients may have had as yet uncharacterized mutations in other genes. This uncertainty may be difficult for patients and their families, particularly if this possibility is not discussed in pre-test counseling.

Presymptomatic and predictive genetic testing or carrier genetic testing of minors is the subject of multiple international guidelines and position papers. Recent systematic reviews of these guidelines [58,59] suggest that in the case of carrier testing of minors, testing should only proceed with proper informed consent. This guideline also applies to potential female carriers of an X-linked disorder and for predictive genetic testing. It is important to stress that such testing can be justified if the results are of direct benefit to the minor, through either access to treatment or to preventative therapy. Thus the testing of asymptomatic males at-risk of XLP could be justified.

The diagnosis of a familial genetic disorder is a potential stressor on family relationships [60]. Parents may report feelings of guilt about passing genetic mutations onto their children. In addition some family members who test negative for the familial mutation may experience survivor guilt.

In summary, the availability of molecular genetic testing has profound implications for patients, their families and their physicians. Genetic counseling plays a critical role in the appropriate use of these tests, which have great potential to improve treatment outcomes for patients.

## Conflicts of interests

The authors declare that they have no competing interests.

## Authors' contributions

RA conceptualized this review and wrote the first draft. This article is based on an invited lecture given to the Royal Australasian College of Pathologists and the World Associations of Pathology and Laboratory Medicine meeting, Sydney 2009.

S-TW undertook most of the laboratory studies described in the paper. She contributed references to the technical aspects of molecular analysis.

KN wrote the discussion section of the paper. She constructed the pedigree of the family with XLP.

DL critically reviewed the manuscript and suggested changes to the final two versions as well as suggesting changes to the references.

All authors have read approved the final manuscript.

## Appendix 1

Advantages of Molecular analysis for PID diagnosis.

Diagnosis

• Distinguishing genetic from acquired disorders

• Confirming the clinical diagnosis

• Identifying novel presentations of PIDs

• Identifying atypical presentations of PIDs

• Urgent diagnosis in infancy where conventional diagnostic tests are unreliable

Treatment

• Assisting treatment decisions

• Gene therapy- identifying those who may benefit from gene based therapy

Prognosis

• Prognosis

Pre-symptomatic testing

• Where presymptomatic diagnosis (at any age) is not possible with protein based tests

• Early identification of disorders which present later in childhood

Screening

• Cascade screening of at-risk relatives

• Population based screening

PID prevention

• Prenatal Diagnosis

• Pre-implantation Genetic diagnosis

Research

• Characterising the role of molecules in cellular function

• Assisting with the classification of primary immunodeficiency disorders

• Identification of new genetic defects
